# CMTM3 protects the gastric epithelial cells from apoptosis and promotes IL-8 by stabilizing NEMO during *Helicobacter pylori* infection

**DOI:** 10.1186/s13099-023-00533-4

**Published:** 2023-02-13

**Authors:** Jing Zhang, Jing Ning, Weiwei Fu, Yanyan Shi, Jing Zhang, Shigang Ding

**Affiliations:** 1 Department of Gastroenterology, Peking University Third Hospital, 49 Huayuan North Road, Haidian District, Beijing, 100191 People’s Republic of China; 2 Research Center of Clinical Epidemiology, Peking University Third Hospital, 49 Huayuan North Road, Haidian District, Beijing, 100191 People’s Republic of China

**Keywords:** *Helicobacter pylori*, CMTM3, IL-8, Apoptosis, NEMO

## Abstract

**Background:**

CKLF-like MARVEL transmembrane domain containing 3 (CMTM3) plays an important role in cancer development. Although *Helicobacter pylori* (*H. pylori*) infection is a main cause of gastric cancer, the function of CMTM3 during *H. pylori* infection remains unclear. CMTM3 expression levels in tissues from *H. pylori-*infected patients and cells co-cultured with *H. pylori* were analyzed. qRT-PCR and ELISA were used to investigate the effects of CMTM3 on interleukin 8 (IL-8) expression. Annexin V/propidium iodide staining was performed to evaluate the function of CMTM3 in the apoptosis of gastric epithelial cells. Proteomic analysis was performed to explore the underlying mechanism of CMTM3 during *H. pylori* infection. The interaction between CMTM3 and NEMO was determined via co-immunoprecipitation, HA-ubiquitin pull-down assay, and immunofluorescence.

**Results:**

*H. pylori* induced a significant increase in CMTM3 expression. CMTM3 inhibited gastric mucosal epithelial cells from apoptosis and increased the expression level of IL-8 during *H. pylori* infection. KEGG pathway enrichment analysis revealed that differentially expressed proteins were involved in epithelial cell signaling in *H. pylori* infection. CMTM3 directly interacted with NEMO, which promoted protein stabilization by down-regulation of its ubiquitylation.

**Conclusions:**

CMTM3 reduces apoptosis and promotes IL-8 expression in the gastric epithelial cells by stabilizing NEMO during *H. pylori* infection. These findings characterize a new role for CMTM3 in host–pathogen interactions and provide novel insight into the molecular regulation of NEMO.

## Background

Over half of the human population is infected with *Helicobacter pylori* (*H. pylori*), a major cause of gastritis, peptic ulcer disease, and gastric cancer [1]. Eradication of *H. pylori* reduces gastric cancer incidence [2]; however, as drug-resistant strains of *H. pylori* are increasing, the eradication rate under triple antibiotic therapy is less than 80% in most countries around the world [3]. The proportion of *H. pylori* annual recurrence is 13% in developing countries [4]. These problems perplex clinicians throughout the world. The immune response plays an effective role in reducing bacterial load; therefore, understanding the host immune response to *H. pylori* is essential for eliminating the pathogen. As the first cell type to interact with *H. pylori* and the main source of the interleukin 8 (IL-8) after *H. pylori* infection, gastric epithelial cells play a critical role in the *H. pylori*-associated immune response [5]. *H. pylori* induces apoptosis in gastric epithelial cells via death receptors in the plasma membrane, which leads to the cleavage of procaspase-8 and release of cytochrome from mitochondria, and activation of subsequent apoptotic events [6].

The nuclear factor κB (NF-κB) pathway, which has a central role in the innate immune response against viral and bacterial infections, increases the expression of IL-8, and promotes cell survival [7, 8]. *H. pylori* with cag pathogenicity island encoding type 4 secretion system, induces the NF-κB pathway [9]. After *H. pylori* infection, IκBα kinase (IKK)—composed of the catalytic subunits IKKα and IKKβ and the scaffold IKKγ/NEMO—activates and phosphorylates IκBα, resulting in IκBα degradation [10–12]. Thereafter, NF-κB dimers translocate to the nucleus and target downstream DNA sequences involved in the inhibition of viruses and bacteria, induction of cytokine and anti-apoptosis proteins expression [13, 14].

CKLF-like MARVEL transmembrane domain containing 3 (CMTM3) belongs to the CMTM family [15, 16]. The CMTM3 gene is a novel gastric cancer suppressor gene that restrains cell migration and invasion [17–21]. However, it’s also reported that CMTM3 has a pro-tumorigenic effect in pancreatic cancer, glioblastoma, and hepatocellular carcinoma. Although the link between *H. pylori* infection and gastric cancer is established, the role of CMTM3 during *H. pylori* infection is unknown.

In this study, we used CMTM3 knockout and overexpress cells to demonstrate the physiological role of CMTM3 and showed that CMTM3 is a positive regulator of the NF-κB pathway upon *H. pylori* infection. CMTM3 interacts specifically with NEMO and mediates ubiquitylation and degradation of NEMO in response to infection. These findings suggest that interaction between CMTM3 and NEMO is essential for maintenance of homeostasis in the immune system.

## Results

### Up-regulation of CMTM3 expression after *H. pylori* infection

**Fig. 1:**
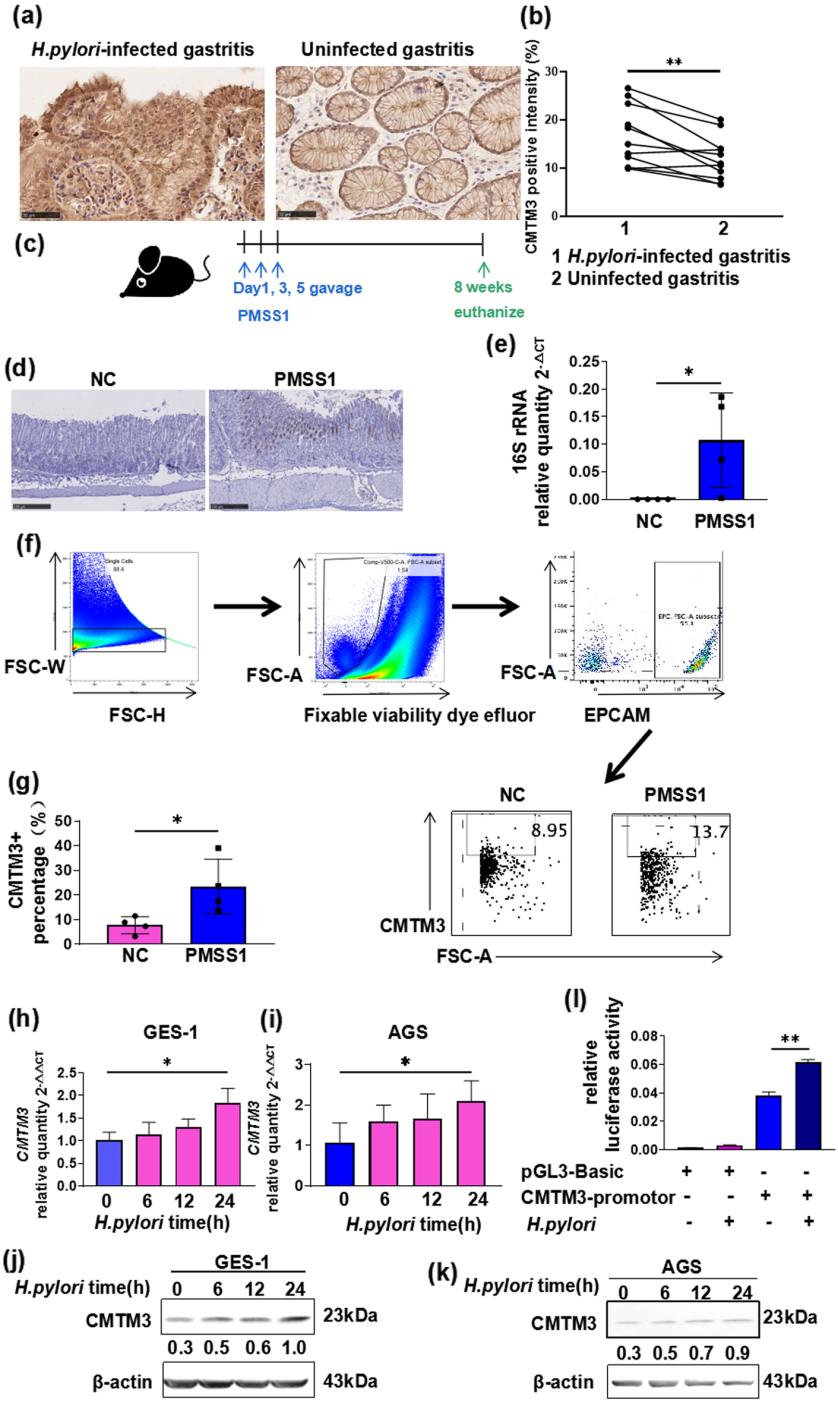
CMTM3 expression increases during *H. pylori* infection. **a** CMTM3 expression level in the gastric mucosa from the same patient before and after *H. pylori* infection (n = 11) were investigated by immunohistochemistry of CMTM3. **b** Graph showing the average quantification of CMTM3-positive cells in at least 6 random fields of one patient. Data are expressed as means ± SEM. **c** Establishment of the C57BL/6 mouse model of *H. pylori* infection. **d&e** *H. pylori* colonization was identified by *H. pylori* immunohistochemical staining **(d)** and 16S rRNA qRT-PCR **(e)**. **f**, **g** The gating strategy for the percentage of CMTM3-positive cells in mice gastric tissue **(f)**. EPCAM is a maker of gastric mucosa epithelial. Total cells (1 × 10^6^) were counted for analysis. The percentage of CMTM3-positive cells in the Brucella broth (negative control, NC) and PMSS1 groups was compared **(g)**. **h–k** CMTM3 expressions in GES-1 and AGS cells, treated with *H. pylori,* were detected by qRT-PCR** (h**, **i)** and western blotting **(j**, **k)**. β-Actin was used as a loading control. Data is representative of three independent experiments and presented as the mean ± SEM. **l** Luciferase assay of GES-1 transfected with control (pGL3-Basic) or pGL3-Basic-CMTM3-promotor plasmid. Renilla vector was used as an internal control plasmid. The relative luciferase activity was calculated as Firefly/Renilla ratio. Data is representative of three independent experiments and presented as the mean ± SEM. *p < 0.05, **p < 0.01

The expression of CMTM3 in the gastric mucosal samples collected before and after *H. pylori* infection from the same patient was determined using immunohistochemistry (Fig. 1a). CMTM3 expression was up-regulated in the *H. pylori*-infected gastritis sample compared to the uninfected ones (Fig. 1b). C57BL/6 mice were challenged with Brucella broth (negative control, NC) or with the PMSS1 strain for 8 weeks (Fig. 1c). *H. pylori* immunohistochemistry staining (Fig. 1d) and 16S rRNA quantitative real-time polymerase chain reaction (qRT-PCR) (Fig. 1e) were used to detect *H. pylori* colonization. EPCAM is a marker of gastric mucosa epithelial cells. We isolated EPCAM-positive cells and analyzed the proportion of CMTM3-positive cells in single cell suspensions from mice gastric tissues using flow cytometry (Fig. 1f). The expression level of CMTM3 *H. pylori*-positive mice was higher than uninfected mice (Fig. 1g).

The change of CMTM3 was verified by *H. pylori*-cell co-culture experiments, in vitro. CagA + VacA + *H. pylori* strain 26,695 infection increased the expression of CMTM3 in GES-1 and AGS cells (Fig. 1h–k). A dual-luciferase activity assay of GES-1cells, transiently transfected with pGL3-Basic-CMTM3-promotor, revealed that *H. pylori* infection induced an increase in CMTM3 at the transcriptional level (Fig. 1l).

### CMTM3 promotes IL-8 production and inhibits apoptosis of gastric epithelial cells during *H. pylori* infection

**Fig. 2:**
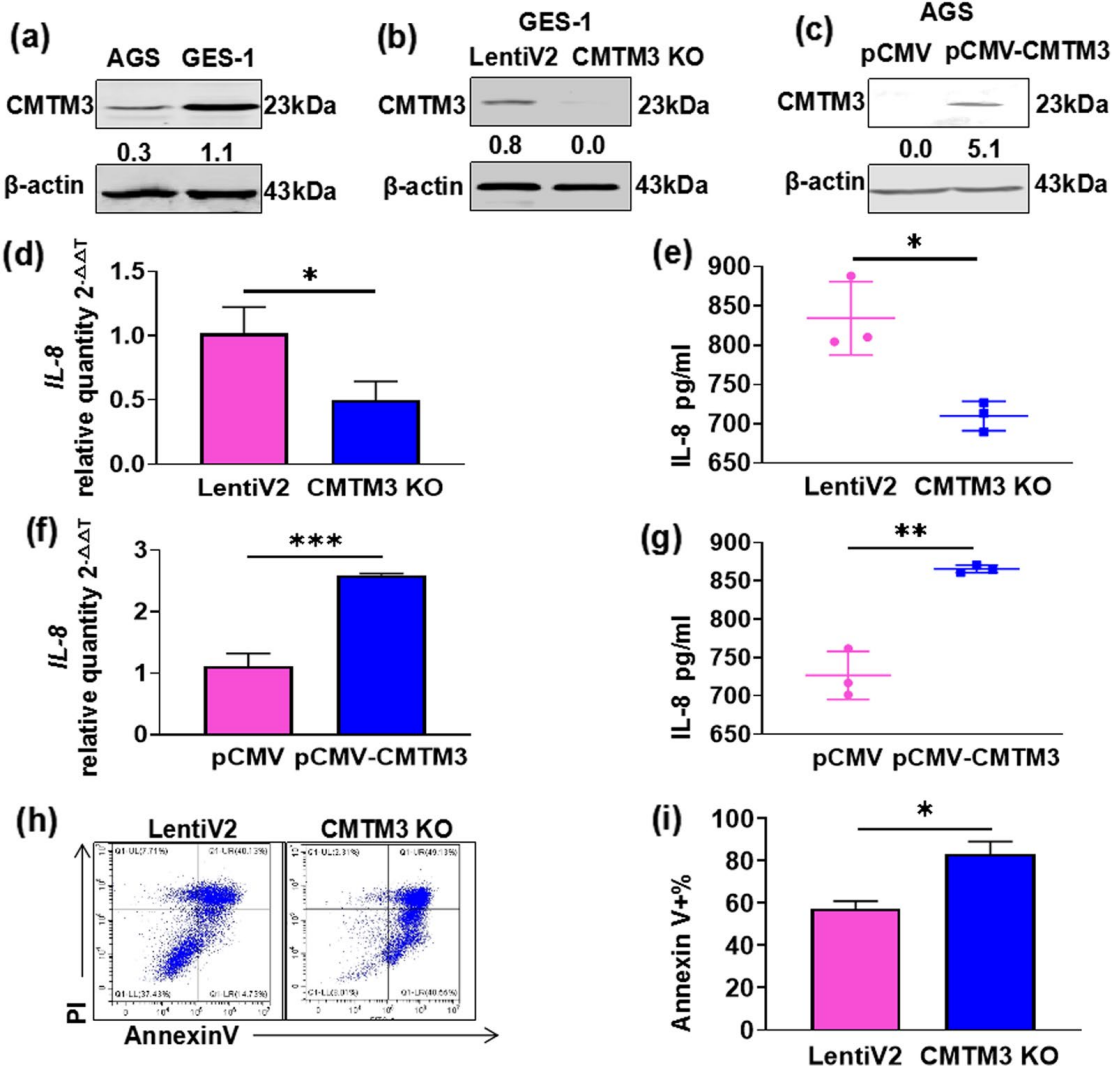
CMTM3 promotes IL-8 expression and inhibits the apoptosis of gastric epithelial cells. **a** CMTM3 expression in GES-1 and AGS cells were analyzed by western blotting. **b** The knockout efficiency of CMTM3 was analyzed by western blotting in GES-1 cells. **c** The overexpression efficiency of CMTM3 was analyzed by western blotting in AGS cells. **d, e** CMTM3 knockout (CMTM3 KO) and control (LentiV2) GES-1 cells were treated with *H. pylori* infection for 24 h. Levels of IL-8 were evaluated by qRT-PCR **(d)** and ELISA **(e)**. **f, g** CMTM3 overexpression (pCMV-CMTM3) and control (pCMV) AGS cells were treated with *H. pylori* for 24 h. Levels of IL-8 were evaluated by qRT-PCR **(f)** and ELISA **(g)**. **h, i** Representative Annexin V/propidium iodide staining of CMTM3 KO and control (LentiV2) GES-1 cells treated with *H. pylori* for 24 h **(h)** and the proportions of Annexin V-positive cells **(i)**. *p < 0.05, **p < 0.01, ***p < 0.001

Some CMTM family members are immunity regulators [22–24]. *H. pylori* induces IL-8 expression in gastric epithelial cells, which is an important source of IL-8 [25]. Therefore, we explored the function of CMTM3 on IL-8. Firstly, we measured the CMTM3 expression level in GES-1 and AGS cells. GES-1 is a immortalized gastric epithelial cell line and AGS is a gastric adenocarcinoma cell line. CMTM3 expression was higher in GES-1 compared to AGS cells (Fig. 2a). Therefore, we knocked out CMTM3 in GES-1 cells (Fig. 2b) and overexpressed CMTM3 in AGS cells (Fig. 2c). We treated CMTM3 knockout (CMTM3 KO) GES-1 cells with *H. pylori* for 24 h and measured IL-8 levels with qRT-PCR and enzyme-linked immunosorbent assay (ELISA). We found that the expression levels of IL-8 were lower in CMTM3 KO GES-1 cells than in control cells (LentiV2) (Fig. 2d, e). IL-8 levels were elevated in CMTM3-overexpressing (pCMV-CMTM3) AGS cells 24 h post-infection, compared to the control cells (pCMV) (Fig. 2f, g). These data indicate that CMTM3 acts as a positive regulator of IL-8 during *H. pylori* infection.

*H. pylori* induces apoptosis of healthy gastric epithelial cells [26]. Annexin V/propidium iodide assay was used to detect apoptosis induced by *H. pylori* in CMTM3 KO GES-1 cells. CMTM3 inhibited *H. pylori*-induced apoptosis 24 h post-infection, compared to the control cells (LentiV2) (Fig. 2h, i).

### Proteomics analysis reveals the role of NEMO in inflammation related to *H. pylori*-CMTM3

**Fig. 3:**
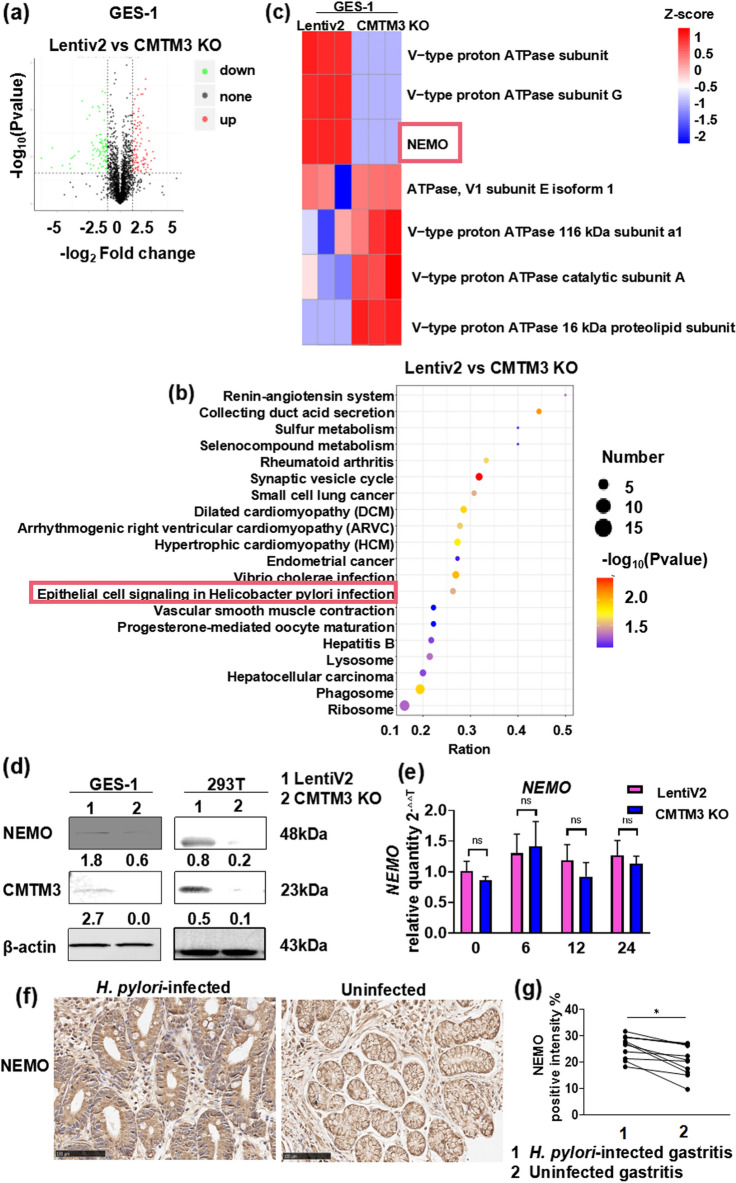
Proteomics analysis indicates that NEMO is involved in *H. pylori*-CMTM3-inflammation. **a** Differentially expressed proteins (P.adj < 0.05). **b** Kyoto Encyclopedia of Genes and Genomes. enrichment analysis. The TOP regulated signaling pathways were shown of CMTM3 KO vs control (LentiV2) in GES-1 cells (padj < 0.05). **c** Heat map of the expression levels of the differentially expressed proteins enriched in epithelial cell signaling in *H. pylori* infection signaling pathway. Red indicates high and blue indicates low protein expression.** d** NEMO expression was detected in CMTM3 KO GES-1 and HEK 293 T cells by western blotting. β-Actin was used as a control. Data are representative of three independent experiments. **e** NEMO expression was detected in CMTM3 KO GES-1 cells by qRT-PCR. Data is representative of three independent experiments. **f** Representative immunohistochemistry images of NEMO by human gastric tissues from the same patient before and after *H. pylori* infection (n = 11). **g** Summary of immunohistochemistry results for NEMO. Graph showing the average quantification of NEMO positive cells in at least 6 random fields of one patient. Data is expressed as means ± SEM. *p < 0.05. ns, no statistical difference

CMTM3 affects many proteins at the post-transcriptional level [21]. Accordingly, we used proteomics technology to identify proteins affected by CMTM3, to explain why CMTM3 promoted IL-8 production and inhibited apoptosis of gastric epithelial cells during *H. pylori* infection. CMTM3 KO GES-1 cells were co-cultured with *H. pylori* (MOI 100:1) for 24 h and evaluated by whole protein label-free proteomics technology. We identified 2187 differentially expressed proteins between CMTM3 KO and LentiV2 GES-1 cells, including 122 up-regulated proteins and 151 down-regulated proteins (fold change > 2; Fig. 3a). Differentially expressed proteins were analyzed using the Kyoto Encyclopedia of Genes and Genomes pathway enrichment analysis. Four differentially expressed proteins, NEMO and three subunits of V-type proton ATPase, were involved in epithelial cell signaling in *H. pylori* infection (Fig. 3b). The heat map showed the Z-score of these proteins (Fig. 3c).

The expression of NEMO, a key molecule in the NF-κB pathway, was decreased following CMTM3 knockout. We confirmed these results via western blotting, revealing that NEMO expression was down-regulated in CMTM3 knockout cells compared to the control cells (LentiV2) (Fig. 3d). However, CMTM3 had no obvious effects on NEMO expression at the mRNA level (Fig. 3e). These results suggested that CMTM3 might contribute to NEMO protein synthesis and degradation.

Using paired gastric tissues from the same patient before and after *H. pylori* infection, we evaluated the protein expression of NEMO by immunohistochemistry. The NEMO protein expression levels during *H. pylori* infection were significantly higher than those of *H. pylori* uninfected patients (Fig. 3f, g).

### CMTM3 promotes IL-8 production and inhibits apoptosis of gastric epithelial cells during *H. pylori* infection by NEMO

**Fig. 4:**
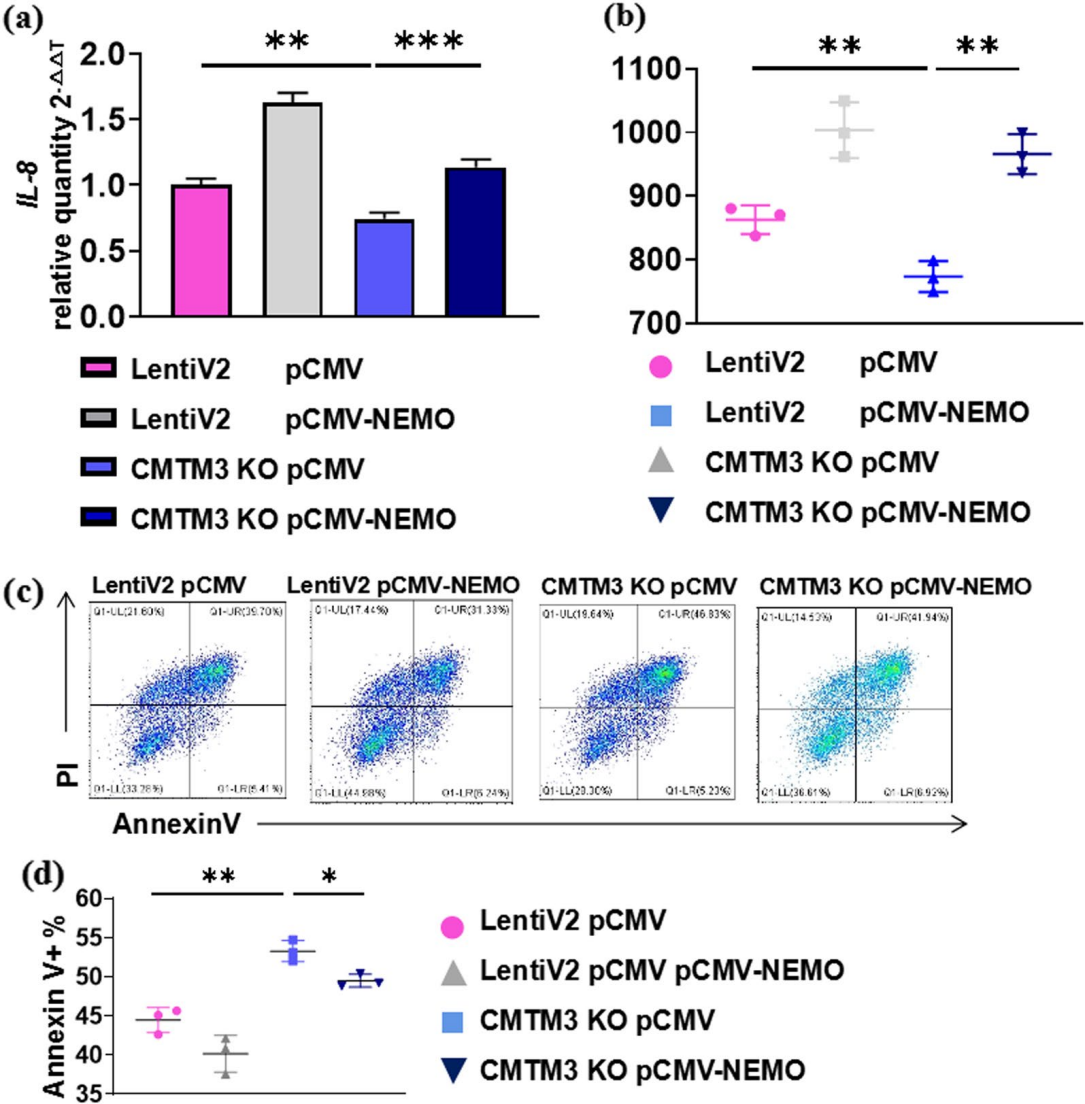
CMTM3 promotes IL-8 production and inhibits the apoptosis of gastric epithelial cells by NEMO. **a, b** The expression level of IL-8 was analyzed by qRT-PCR **(a)** and ELISA **(b)**. After overexpression of NEMO (pCMV-NEMO) in CMTM3 KO GES-1 cells for 24 h, cells were treated with *H. pylori* for 24 h. pCMV was the control vector. Data is representative of three independent experiments. **c, d** Representative Annexin V/propidium iodide staining results **(c)**. After overexpression of NEMO (pCMV-NEMO) in CMTM3 KO GES-1 cells for 24 h, cells were treated with *H. pylori* for 24 h. pCMV was the control vector. Annexin V-positive cells were analyzed** (d)**. Data is representative of three independent experiments. *p < 0.05, **p < 0.01, ***p < 0.001

To explore that CMTM3 promotes IL-8 production and inhibits apoptosis of gastric epithelial cells were dependent on NEMO, we overexpressed NEMO in CMTM3 KO cells. The IL-8 level was higher in pCMV-NEMO CMTM3 KO cells than the control (pCMV) CMTM3 KO cells on the mRNA (Fig. 4a) and protein level (Fig. 4b). These results suggested CMTM3 promoted IL-8 production during *H. pylori* infection by NEMO.

We overexpressed NEMO in CMTM3 KO cells. The proportion of apoptotic cells decreased in pCMV-NEMO CMTM3 KO cells than control (pCMV) CMTM3 KO cells (Fig. 4c, d). These results suggested CMTM3 inhibited apoptosis of gastric epithelial cells during *H. pylori* infection by NEMO.

### CMTM3 reduces NEMO degradation

**Fig. 5:**
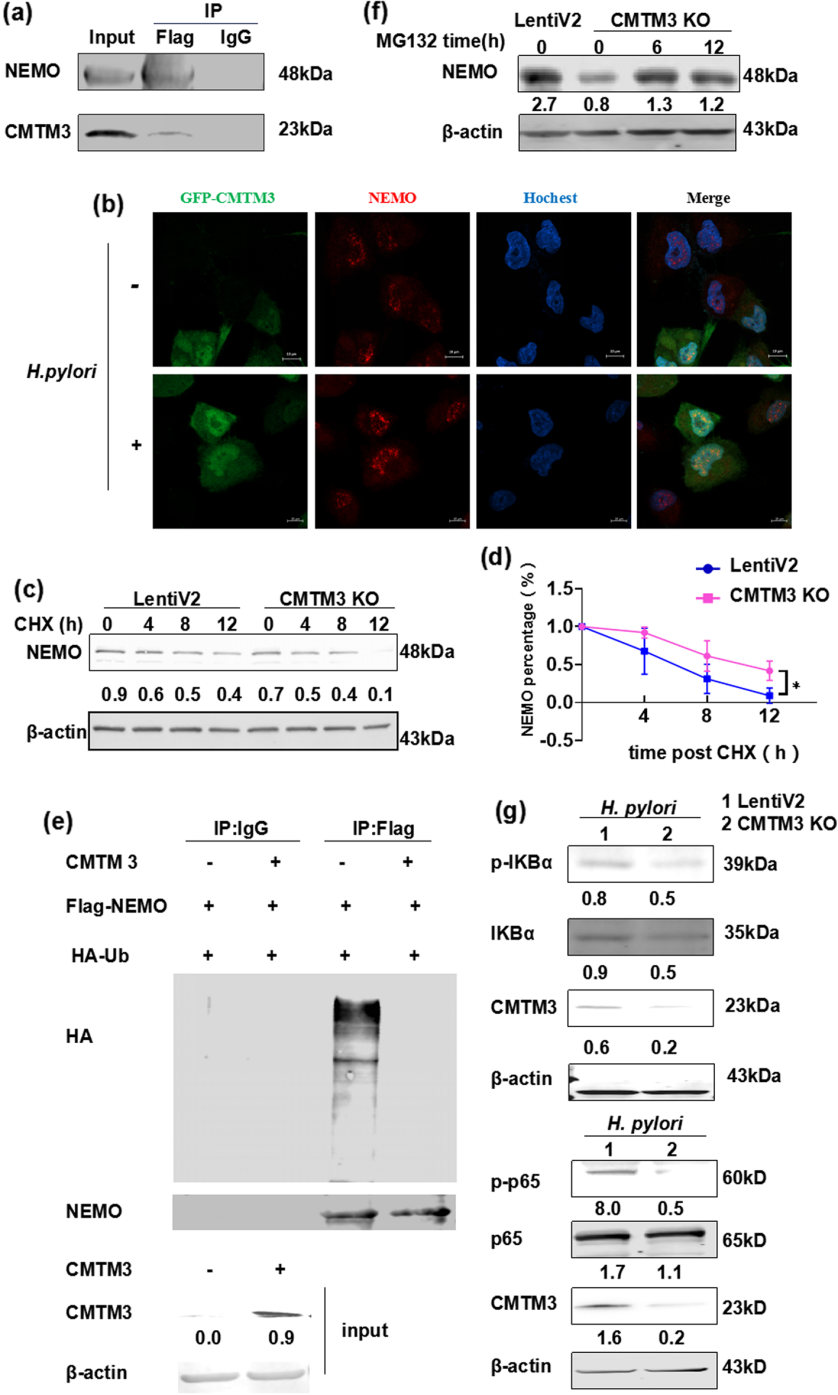
CMTM3 reduces NEMO degradation. **a** The immunoprecipitation analysis of GES-1 cells. Cells expressing pCMV-NEMO-3Flag were immunoprecipitated by an anti-Flag antibody and immunoblotted by anti-NEMO and anti-CMTM3 antibodies. **b** Co-localization of CMTM3 and NEMO in GES-1 cells was investigated by immunofluorescence co-localization analysis after *H. pylori* infection for 24 h. Scale bar, 10 μm. Hochest was used for nuclear staining. **c, d** CMTM3 knockout GES-1 cells were treated with cycloheximide (25 µg/mL) and proteins were subjected to immunoblotting **(c)**. Data is representative of three independent experiments. The remaining NEMO was quantified **(d)**. **e** The ubiquitylation of NEMO. HEK 293 T cells were transfected with the indicated vectors. After 48 h transfection, cells were treated with MG132 (10 μM) for 6 h. Then cells were lysed and immunoprecipitated with anti-Flag antibody and analyzed by immunoblotting with the indicated antibodies. **f** CMTM3 KO GES-1 cells were treated with MG132 (10 μM) and collected at indicated time points. Whole-cell lysates were subjected to immunoblotting with indicated antibodies. **g** The expression the phosphorylated IKBα (p-IKBα) and phosphorylated p65 (p-p65) were analyzed in CMTM3 KO GES-1 cells.*p < 0.05

To further explore the detailed molecular mechanism between CMTM3 and NEMO, we expressed Flag-tagged NEMO in GES-1 cells and conducted a Flag pulldown assay. We found that CMTM3 interacted with NEMO (Fig. 5a). Additionally, we confirmed co-localization of CMTM3 and NEMO. As shown in Fig. 5b, co-localization of CMTM3 and NEMO occurred in the nucleus.

Thus, we assessed the functions of CMTM3 on NEMO degradation using cycloheximide, which could bind to the 80S ribosomes to inhibit protein biosynthesis in eukaryotes. As shown in Fig. 5c, d, NEMO degradation was accelerating in CMTM3 KO cells compared with control (LentiV2) cells. Altogether, CMTM3 upregulated NEMO expression by inhibiting NEMO degradation in GES-1 cells.

ubiquitylation, a kind of protein modification, is crucial for NEMO degradation. We discovered that CMTM3 inhibited total ubiquitylation level of NEMO (Fig. 5e). Next, we treated GES-1 cells with proteasomal inhibitor MG132 to investigate the possibility that NEMO is regulated by CMTM3. Expression of NEMO was recovered in the presence of MG132 (Fig. 5f), suggesting that CMTM3 regulated the proteasomal degradation of NEMO.

As CMTM3 reduced NEMO degradation, we detected the effect of CMTM3 KO in IKBα and p65 phosphorylation which reflected the NF-κB pathway activity. Upon *H. pylori* treatment, IKBα and p65 phosphorylation decreased iKO cells compared with control (LentiV2) cells (Fig. 5g). Therefore, these data suggest that CMTM3 may promote the NEMO/NF-kB pathway.

## Discussion

*H.pylori* is related to peptic ulcer disease and gastric cancer [27–29]. Although most people develop asymptomatic infections, the eradication of *H. pylori* reduces the risk of gastric cancer in asymptomatic individuals [30]. But the eradication rate under triple antibiotic therapy is less than 80% in most countries [3]. Therefore, treating *H. pylori* infection is problematic.

Many studies have demonstrated that CMTM3 contributes to cancers and acts as a gastric cancer suppressor [20, 21]. However, the role of CMTM3 during *H. pylori* infection, representing an early stage in the development of gastric cancer, is unclear. Here, we revealed that CMTM3 increased in the acute *H. pylori* infection and promoted IL-8 expression, suggesting that CMTM3 may play an important role in the inflammatory response to the *H. pylori*. More and more members of the CMTM family have been reported to participate in the immune response. CMTM6 and CMTM4 reduce the ubiquitylation of PD-L1 and increase its protein half-life, which affects tumor immune responses [22, 23]. CMTM4 is also identified as a component of IL-17R and mediates autoimmune diseases [24]. This work characterizes a new role of CMTM3 as an immunity regulator during *H. pylori* infection.

A whole genome analysis of the gastric epithelial cells response to *H. pylori* exposure reveals that IL-8 is the most markedly up-regulated gene [31]. IL-8 recruits neutrophils and lymphocytes to the infected gastric tissue to eliminate *H. pylori* in the early stage of infection. IL-8 is also involved in Th17 cell differentiation [32, 33]. Th17 is involved in protection against *H. pylori* via IL-17 secretion, which is important for eliminating *H. pylori* [34]. However, extremes are dangerous. The milieu of excessive chronic inflammatory reaction increases the risk of neoplastic changes**.** It is reported that IL-8 is up-regulated in many cancers and has tumourigenic effects by long-term stimulating angiogenesis, neutrophil recruitment and proliferation and migration of tumour cells [35–37]. However, it takes a long time for from inflammation to tumor. GES-1 cells are co-cultured with *H. pylori* for 45 days and present with the characteristics of intestinal-type gastric cancers in vitro [38]. In another study, treating cells with *H. pylori* repeatedly every 24 h for approximately 8 weeks is considered as a chronic infection model [39]. In our study, the cell-*H. pylori* coculture for 24 h may only represent the conditon of an acute inflammatory. Moreover, we should notice that only a few *H. pylori*-infected individuals will develop a chronic inflammatory response, suggesting that there are precise regulatory mechanisms for the inflammation. Next, We plan to generate CMTM3 KO mouse models to study the long-term role of CMTM3 in the development of *H. pylori-*related diseases.

*H. pylori* strongly adheres to gastric cells and causes diverse cell damage via its major cytotoxins, and proteins. We have previously shown that *H. pylori* considerably induces apoptosis in gastric epithelial cells at the early stage of infection [26]. *H. pylori* induces the apoptosis of gastric epithelial cells to reduce the production of gastric acid secretion in the stomach, which is benefit for its gastric colonization [40]. Elevated apoptosis correlates with increased disintegration of cell monolayers, which destroys the local homeostasis [41]. According to the report, IL-33 can promote the restoration of gastric cell homeostasis, due to the inhibition apoptosis [42, 43]. Besides, gastric epithelial cells are part of gastric mucosal barrier, which integrity is crucial for the prevention of *H. pylori-* related gastric diseases [44]. In this study, we discovered that CMTM3 inhibits the *H. pylori*-induced apoptosis of gastric epithelial cells, which may affect the integrity of the gastric mucosal epithelial barrier and *H. pylori* colonization.

NEMO, a crucial scaffolding molecule in the activation of NF-κB, was identified in the proteomics analysis (LentiV2 vs. CMTM3 KO GES-1 cells) [45]. The NF-κB pathway induces the expression of cytokines and anti-apoptosis proteins in gastric epithelial cells, which is critical for the immune response and cell survival [46]. In hepatocytes, NEMO physiologically inhibits apoptosis by regulating NF-κB activation and also preventing the formation of the complex RIPK1- FADD- Caspase-8 [47, 48]. TRIM14 induces IL-8 expression by directly binding to NEMO and promotes the phosphorylation of IKBα and p65 [49]. Many pathogens, such as SARS-CoV-2, *Brucella melitensis,* and *Shigella*, disrupt NF-κB signaling and immune responses by targeting NEMO during infection, resulting in persistent infection [50–52]. In this study, we discovered that CMTM3 inhibited the *H. pylori*-induced apoptosis and promoted the IL-8 expression in gastric epithelial cells. CMTM3 interacts directly with NEMO in the nucleus under *H. pylori* treatment. It has been reported that NEMO can be ubiquitinated in the nucleus under genotoxic stress, which is a key step in the activation of NF-κB [53]. DNA damage activates IKK-unbound NEMO in the nucleus via SUMO (small ubiquitin-like modifier) -1 attachment and ATM-dependent ubiquitylation. Then NEMO shifts from the nucleus to the cytoplasm to activate IKK [54]. TRIM37, a novel E3 ligase, plays a vital role in the nuclear export of NEMO and IKK/NF-κB activation via ubiquitylation of NEMO in the nucleus [55]. Therefore, we speculated that CMTM3 regulated the ubiquitylation of NEMO in the nucleus, thereby affecting NEMO degradation and NF-κB activation [56]. While we characterized the CMTM3 inhibited total ubiquitylation level of NEMO, further research is needed to identify the type of NEMO ubiquitylation regulated by CMTM3 and its underlying mechanism.

Long-term asymptomatic *H. pylori* infection is associated with an increased risk for the development of *H. pylori*-related diseases. CMTM3 is up-regulated during *H. pylori* infection, which promotes IL-8 production and inhibits the apoptosis of gastric epithelial cells. Accordingly, the appropriate expression of CMTM3 may play a vital role in the immunity for the eradication of *H. pylori*.

## Conclusion

**Fig. 6:**
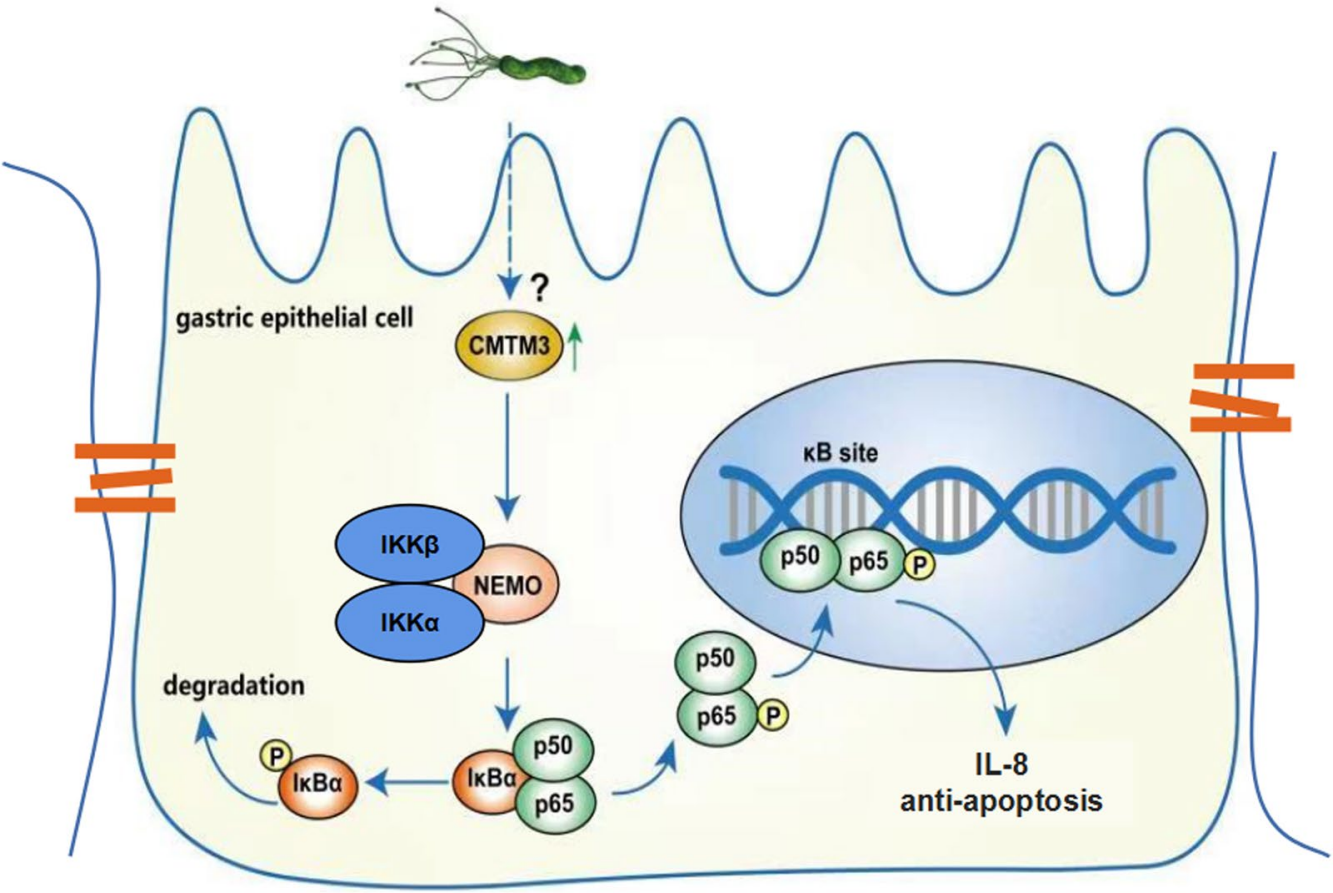
Schematic diagram of CMTM3 function during *H. pylori* infection. *H. pylori* induces CMTM3 expression, and CMTM3 promotes IL-8 production and inhibits the apoptosis of gastric epithelial cells by stabilizing NEMO

CMTM3 reduces apoptosis and promotes IL-8 expression in the gastric epithelial cells by stabilizing NEMO during *H. pylori* infection. These findings characterize a new role for CMTM3 in host–pathogen interactions and provide novel insight into the molecular regulation of NEMO (Fig. 6).

## Methods

### Tissues and immunohistochemistry

Human gastric mucosal tissues were collected from the Department of Digestive Pathology of the Peking University Third Hospital (Beijing, China). Eleven paired samples of the gastric mucosa were obtained from the same patient who was infected with *H. pylori* and successfully eradicated *H. pylori* for more than one year. Because *H. pylori* can adsorb anions and appear black, Warthin-Starry staining was used to confirm *H. pylori* infection. If there is *H. pylori* infection, there are helix rods in the gastric mucosa under Warthin-Starry staining [57]. CMTM3 and NEMO immunohistochemistry were performed as previously described, following the instructions of the antibodies [58]. Six fields were randomly selected for analysing by ImageJ 1.52a software (Silver Spring, Maryland, USA). Average number of the positive area was compared by paired t-tests. The Ethics Committee of the Peking University Third Hospital approved the study (decision numbers S2022179).

### Antibodies

Detailed information regarding the antibodies used in this study is provided in Table 1.

**Table 1:** Detailed information about antibodies

Name	Vendor	Catalog number	Application	City	Country
Anti-CMTM3	Affinity	DF3943	WB	Liyang	China
Anti-NEMO	Abcam	ab178872	WB, IHC	Cambridge	UK
Anti-β-Actin	Proteintech	66009-1	WB	Wuhan	China
Phospho-p65	Immunoway	YP0191	WB	California	USA
p65	Proteintech	66535-1-Ig	WB	Wuhan	China
IKBα	Abmart	T55026	WB	Shanghai	China
Phospho-IKBα	Abmart	TP56280	WB	Shanghai	China
Fixable viability dye efluor	Invitrogen	65-0866-18	FCM	Shanghai	China
Anti-CMTM3	Sigma	HPA013870	IHC	Shanghai	China
Anti-flag	Thermo	14-6681-80	Co-IP	Shanghai	China
Anti-HA	Proteintech	66006-2-Ig	WB	Wuhan	China
Mouse IgG isotype	Proteintech	B900620	Co-IP	Wuhan	China
TRITC-anti-rabbit	ZSGB-BIO	ZF-0136	IF	Beijing	China

### Animals

Six- to eight-week-old male C57BL/6 mice were obtained from the Laboratory Animal Science of Peking University (Beijing, China). The mice were divided into 2 groups, the control (NC) and *H. pylori-*infection (PMSS1) group. *H. pylori* strain PMSS1 was utilized in this study [39]. Briefly, after fasting for 24 h, mice in PMSS1 and NC groups were intragastrically administered 2.5 × 10^9^ CFU PMSS1 and isometric Brucella broth respectively every other day for 3 times. After 8 weeks of infection, the mice were sacrificed [40]. The Ethics Committee of the Peking University Third Hospital authorized the animal research (decision numbers SA2022156).

### *H. pylori* strains

*H. pylori* strain PMSS1 was a kind gift from the Chinese Center for Disease Control and Prevention. *H. pylori* 26695 was maintained in our laboratory. *H. pylori* strain was verified by *H. pylori* urease test kit (Begen, Sanming, China), Gram staining, and sequencing results. *H. pylori* urease test kit is based on that *H. pylori* secretes a large amount of highly active urease which can catalyze urea and produce ammonia. The pH value increases and causes the indicator to change into red. *H. pylori* infection can be determined according to the color change [59]. Besides, *H. pylori* is a gram-negative bacillus which is a blue curved rod under the gram staining. *H. pylori* was cultured following standard protocol [58]. Briefly, *H. pylori* was cultured on Karmali solid medium containing 10% sterile defibrinated sheep blood. Every two days, the *H. pylori* was passaged*. H. pylori* was cultured under micro-aerobic conditions (10% CO_2_, 85% N_2_, 5% O_2_, and 37 °C).

## Gastric epithelial single cell suspension and flow cytometry

Briefly, the stomachs of the mice in each group were removed and washed with PBS. The glandular stomachs were cut and incubated in 5 mM EDTA for 2 h at 4 °C with gentle shaking. Then, isolated glands were passed through a 70 µm nylon mesh cell strainer, and centrifuged at 150 × *g* for 5 min [60]. Surface antigen stainings were performed using antibodies against CMTM3 (kindly provided by Prof. Han Wenling) and Fixable Viability Dye eFluor on ice for 30 min. The percentage of CMTM3 positive cells was analyzed using a BD flow cytometer (Franklin Lake, New Jersey, USA) and results were analyzed by FlowJo 10.8.1 (Franklin Lake, New Jersey, USA).

### Cell culture and infection

Human cell lines HEK 293 T, and AGS were maintained from ATCC (Manassas, Virginia, USA). Human cell line GES-1 was from Shanghai Institute of Cell Biology, Chinese Academy of Sciences (Shanghai, China). GES-1 and AGS cells were cultured in Roswell Park Memorial Institute 1640 medium (HyClone, Logan, Utah, USA) containing 10% fetal bovine serum (Gibco, Waltham, Massachusetts, USA), and 100 U/mL penicillin–streptomycin (Gibco). HEK 293 T cells were cultured in DMEM (HyClone) supplemented with 10% fetal bovine serum and 100 U/mL penicillin–streptomycin. Cells were cultured at 37 °C with 5% CO_2_.

The *H. pylori*-cell co-culture experiment was carried out as described previously [61]. Briefly, cells were seeded in proper cell culture plates or dishes. After 12 h, *H. pylori* was collected from the solid medium, washed, and resuspended in PBS. The quantity of *H. pylori* was measured at 530 nm absorbance using a spectrophotometer. The *H. pylori* was added to the culture medium of cells at the multiplicity of infection (MOI) 100:1 at 37 °C with 5% CO_2_, without penicillin–streptomycin.

### Western blotting

Cells were washed with cold PBS and then lysed by RIPA buffer (Solarbio, Beijing, China) with protease and phosphatase inhibitors (Solarbio) on ice for 30 min. The protein concentration was estimated using the BCA Protein Assay Kit (Thermo Fisher Scientific, Waltham, Massachusetts, USA). For SDS-PAGE and immunoblotting, samples were heated at 99 °C for 10 min, separated on Tris–Glycine gels at the constant condition of 30 mA, and transferred onto membranes (Millipore, Billerica, Massachusetts, USA) at the constant condition of 100 V for 2 h. After blocking with 5% nonfat milk or 5% bovine serum albumin for 1 h at room temperature (RT), the membranes were incubated with the first antibodies for 16 h at 4 ℃ and the second antibodies for 1 h at RT. Enhanced chemiluminescence reagent (Yamei, Shanghai, China) or the Odyssey detection system (LI-COR, Lincoln, Nebraska, USA) were used to detect protein expression. Gray values obtained by western blotting were analyzed by ImageJ 1.52a software.

### Generation of CMTM3 knockout cells

For the generation of CMTM3 knockout (CMTM3 KO) cells, HEK 293 T or GES-1 cells were infected with a lentivirus (lentiCRISPR v2) encoding a guide RNA (gRNA) (5′-CCTGCCGGCGGCTCCCGTCCCGG-3′). The gRNA was designed via Zhang Lab (https://zlab.bio/guide-design-resources). The lentiviruses were produced by HEK 293 T cells with three vectors, lentiCRISPRv2-gRNA, pCMV-VSV-G, and psPAX2. Cells were infected with the lentivirus containing lentiCRISPRv2-gRNA. The CMTM3 stable knockout cells were screened out by puromycin (Yuanye, Shanghai, China).

### Plasmids and cell transfection

The plasmid pCMV-MCS-IKBKG-3FLAG (pCMV-NEMO-3FLAG) was bought from Yibaike, (Beijing, China). pCMV-CMTM3 was a kind gift from Prof. Wenling Han. Cells were transfected with plasmids using Lipofectamine 2000 transfection reagent (Invitrogen, Shanghai, China) and Opti-MEM (Gibco). After the transfection for 24 h, cells were applied to *H. pylori*-cell co-culture.

### siRNA transfection

To knock down endogenous NEMO, small interfering RNA (siRNA) constructs were purchased from RIBOBIO (Guangzhou, China; genOFFTM st-h-IKBKG_001:5′-AGTTGCAGGTGGCCTATCA-3′). AGS cells were transfected with siRNA at a final concentration of 50 nM, using RFect siRNA Transfection Reagent (Baidai, Changzhou, China). After the transfection for 48 h, cells were applied to *H. pylori*-cell co-culture.

### qRT-PCR

The total RNA was extracted by TRIzol (Invitrogen). Reverse transcription and qRT-PCR were performed using the Fast-King RT Kit and qPCR Mix SYBR Green Kit (Tiangen Biotech, Beijing, China). Amplifications were performed according to the manufacturer’s instructions. Sequences of quantitative qRT-PCR primers are mentioned in Table 2. *β-ACTIN* was used as a control. Relative mRNA expression levels of *IL-8* and *H. pylori* 16S rRNA were determined using the 2^−∆∆Ct^ method.

**Table 2:** Sequences of qRT-PCR primers

Gene name	Sequence
*β-ACTIN *	(F)5′-CATGTACGTTGCTATCCAGGC-3′
	(R)5′-CTCCTTAATGTCACGCACGAT-3′
*H.pylori 16S rRNA*	(F)5′GCTCGTGTCGTGAGATGTT3′
	(R)5′ACGGAGGCAGTATCCTTAGA3′
*IL-8*	(F)5′-ACTGAGAGTGATTGAGAGTGGAC-3′
	(R)5′-AACCCTCTGCACCCAGTTTTC-3′

### Apoptosis assay

GES-1 cells were seeded in 12-well plates at a density of 2 × 10^5^. After co-culture with *H. pylori* (MOI 100:1) for 24 h, the culture medium and cells were collected and washed with PBS. An Annexin V/propidium iodide Apoptosis Detection Kit (Dojindo, Shanghai, China) was used according to the instruction of the manufacturer. The samples were analyzed by flow cytometry (Beckman, Bria, California, USA). A blank and positive control were made. Apoptosis was induced as a positive control at 55 ℃ for 2 min.

### Proteome and data analysis

CMTM3 KO GES-1 cells were seeded at a density of 4 × 10^6^ cells per 10 cm dish. After 12 h, GES-1 cells were co-cultured with *H. pylori* (MOI 100:1) for 24 h. Cells were washed with PBS, collected, and preserved at − 80 °C for the label-free proteome analysis by Novogene Co. Ltd. (Beijing, China).

Proteins were quantified by Bradford protein quantitative kit. 20 μg protein sample was loaded to 12% SDS-PAGE gel. The concentrated gel was carried out at 80 V for 20 min, the separation gel at 120 V for 90 min. Then, the gel was stained by Coomassie Brilliant Blue R-250 and decolored. Protein samples were treated with DB lysis buffer, trypsin, and TEAB buffer at 37 °C for 4 h. Then trypsin and CaCl_2_ were added to digest the proteins overnight. Formic acid was added to the digested samples and centrifuged at 12,000 × *g* for 5 min, at RT. The supernatant was slowly loaded to the C18 desalting column and washed 3 times with washing buffer, then added to an elution buffer. The eluents of each sample were collected and lyophilized. The powder was dissolved in 10 μL of solution A (100% water, 0.1% formic acid), and centrifuged at 14,000 × *g* for 20 min at 4 ℃. 1 µg peptide were separated and analyzed by Q ExactiveTM HF-X mass spectrometer (Thermo Fisher Scientific). All spectra were searched against the homo_sapiens_uniprot_2021_7_15.fasta (202,195 sequences) database, using Proteome Discoverer 2.2 (PD 2.2; Thermo).

Differentially expressed proteins, with an adjusted p-value (padj) < 0.05 and a fold change > 2, were considered as differentially expressed, as shown by the volcano map. The Cluster Profiler R package was used to test the statistical enrichment of differentially expressed proteins in the Kyoto Encyclopedia of Genes and Genomes database. The difference was considered significant at padj < 0.05. Proteins of interest were analyzed by Z-score and presented in a heat map.

### Immunoprecipitation

GES-1 cells, transfected with pCMV-MCS-IKBKG-3FLAG for 48 h, were used for immunoprecipitation. The cells were collected and lysed by immunoprecipitation RIPA buffer (Solarbio) with protease and phosphatase inhibitors, on ice for 30 min. The whole cell lysate (2 mg) was used for immunoprecipitation with 8 µg anti-flag or mouse IgG isotype control antibody for 16 h at 4 °C with rotation. Then, 50 µL of pre-cleared Protein G Sepharose beads (Thermo Fisher Scientific) were added to the antibody-antigen complex and incubated for another 4 h at 4 °C. The bead-antibody-antigen compound was washed 5 times and analyzed by western blotting.

### NEMO degradation assay

GES-1 KO cells were treated with 25 μg/mL cycloheximide (Selleck, Shanghai, China). At the 0, 4, 8, 12 h, cells were washed with PBS and collected. The remaining NEMO was measured by western blotting, as described above.

### Immunofluorescence

GES-1 cells were seeded on a glass culture dish. The cells were fixed with paraformaldehyde for 15 min. After the cells were permeabilized by 1% TritonX-100 for 15 min at RT, cells were blocked with 5% bovine serum albumin for 1 h at RT. Cells were then incubated with the primary antibodies at 4 °C for 16 h, washed, and incubated with the secondary antibodies for 1 h at RT. Cells were stained with Hoechst 33,342 (Beyotime, Beijing, China) for 10 min at RT. Images were acquired by a Zeiss confocal microscope (Oberkochen, Badenwueberg, Germany).

### Luciferase reporter assay

GES-1 cells were seeded in 12-well plates at a density of 2 × 10^5^ cells for 16 h. GES-1 cells were transfected with 1 µg pGL3-Basic-CMTM3-promotor vector or 1 µg pGL3-Basic vector and 1 µg thymidine kinase promoter-Renilla (TK-Renilla)luciferase reporter vector, using 4 µl Lipofectamine2000. pGL3-Basic-CMTM3-promotor vector and TK-Renilla vector were bought from Sangon (Shanghai, China). After 24 h transfection, cells were treated with *H. pylori* (MOI 100:1) for 24 h. Luciferase activity was then determined, using a dual-luciferase reporter assay system (Meilunbio, Dalian, China) following the instruction of the manufacturer. Renilla vector was measured as a control. The relative luciferase activity was calculated as Firefly/Renilla ratio.

### HA-ubiquitin pull-down assay

HEK 293 T cells were seeded in 10 cm dishes at a density of 4 × 10^6^ for 16 h and transfected with 4 µg pCMV-NEMO-3FLAG, 4 µg Ha-Ub (Yibaike, Beijing, China), and 4 µg pCMV-CMTM3, or 4 µg pCMV vector, using 24 µl Lipofectamine2000. After 48 h transfection, cells were treated with 10 μM MG132 (Selleck) for 6 h. Thereafter, the cells were washed with cold PBS, collected, and lysed by immunoprecipitation RIPA buffer (Solarbio) with protease and phosphatase inhibitors (Solarbio) and N-ethyl malebutylene diimide (Meilun, Dalian, China), on ice for 30 min. 2 mg of whole cell lysate was used for immunoprecipitation with 8 µg anti-Flag antibody or mouse IgG isotype control antibody overnight at 4 °C with rotation. Then, 50 µL pre-cleared Protein G Sepharose beads (Thermo) were added to the antibody-antigen complex and incubated for another 4 h at 4 °C. The bead-antibody-antigen compound was washed 5 times with immunoprecipitation RIPA buffer, containing protease and phosphatase inhibitors and N-ethyl malebutylene diimide buffer. The samples were loaded onto a 7.5% SDS-PAGE gel and analyzed by western blotting.

### Statistical analysis

Statistical analyses were performed using SPSS software 27.0 (Chicago, Ilinois, USA). Student's t-test or ANOVA was used to evaluate significant differences. p < 0.05 was considered statistically significant.

## Data Availability

All data generated or analyzed during this study are included in this published article.

## References

[1] Hooi J, Lai WY, Ng WK, Suen M, Underwood FE, Tanyingoh D (2017). Global prevalence of *Helicobacter pylori* infection: systematic review and meta-analysis. Gastroenterology.

[2] Hu Y, Wan JH, Li XY, Zhu Y, Graham DY, Lu NH (2017). Systematic review with meta-analysis: the global recurrence rate of *Helicobacter pylori*. Aliment Pharmacol Ther.

[3] Zhang M (2015). High antibiotic resistance rate: a difficult issue for *Helicobacter pylori* eradication treatment. World J Gastroenterol.

[4] Niv Y (2008). *H. pylori* recurrence after successful eradication. World J Gastroenterol.

[5] Morey P, Pfannkuch L, Pang E, Boccellato F, Sigal M, Imai-Matsushima A (2017). *Helicobacter pylori* depletes cholesterol in gastric glands to prevent interferon gamma signaling and escape the inflammatory response. Gastroenterology.

[6] Ashktorab H, Dashwood RH, Dashwood MM, Zaidi SI, Hewitt SM, Green WR (2008). *H. pylori*-induced apoptosis in human gastric cancer cells mediated via the release of apoptosis-inducing factor from mitochondria. Helicobacter.

[7] Bellet MM, Pieroni S, Castelli M, Piobbico D, Fallarino F, Romani L (2020). HOPS/Tmub1 involvement in the NF-kB-mediated inflammatory response through the modulation of TRAF6. Cell Death Dis.

[8] Wan XK, Yuan SL, Tao HX, Diao LP, Wang YC, Cao C (2016). The upregulation of TRAF1 induced by *Helicobacter pylori* plays an antiapoptotic effect on the infected cells. Helicobacter.

[9] Ferrand J, Lehours P, Schmid-Alliana A, Megraud F, Varon C (2011). *Helicobacter pylori* infection of gastrointestinal epithelial cells in vitro induces mesenchymal stem cell migration through an NF-kappaB-dependent pathway. PLoS ONE.

[10] Maubach G, Schm Dicke AC, Naumann M (2017). NEMO links nuclear factor-κB to human diseases. Trends Mol Med.

[11] May MJ, D'Acquisto F, Madge LA, Gloeckner J, Pober JS, Ghosh S (2000). Selective inhibition of NF-κB activation by a peptide that blocks the interaction of NEMO with the IκB kinase complex. Science.

[12] Sun S, Ganchi P, Ballard D, Greene W (1993). NF-kappa B controls expression of inhibitor I kappa B alpha: evidence for an inducible autoregulatory pathway. Science.

[13] Xz A, Lz A, Ll A, Zz A, Bo ZA, Jy A (2020). MxA suppresses TAK1-IKKα/β-NF-κB mediated inflammatory cytokine production to facilitate Mycobacterium tuberculosis infection. J Infect.

[14] Zhuo Y, Guo Z, Ba T, Zhang C, He L, Zeng C (2021). African swine fever virus MGF360-12L inhibits type I interferon production by blocking the interaction of importin alpha and NF-kappaB signaling pathway. Virol Sin.

[15] Han W, Ding P, Xu M, Wang L, Rui M, Shi S (2003). Identification of eight genes encoding chemokine-like factor superfamily members 1–8 (CKLFSF1-8) by in silico cloning and experimental validation. Genomics.

[16] Han W, Lou Y, Tang J, Zhang Y, Chen Y, Li Y (2001). Molecular cloning and characterization of chemokine-like factor 1 (CKLF1), a novel human cytokine with unique structure and potential chemotactic activity. Biochem J.

[17] Su Y, Lin Y, Zhang L, Liu B, Yuan W, Mo X (2014). CMTM3 inhibits cell migration and invasion and correlates with favorable prognosis in gastric cancer. Cancer Sci.

[18] Yuan W, Liu B, Wang X, Li T, Xue H, Mo X (2017). CMTM3 decreases EGFR expression and EGF-mediated tumorigenicity by promoting Rab5 activity in gastric cancer. Cancer Lett.

[19] Chrifi I, Louzao-Martinez L, Brandt M, Dijk CV, Burgisser P, Zhu C (2017). CMTM3 (CKLF-like marvel transmembrane domain 3) mediates angiogenesis by regulating cell surface availability of VE-cadherin in endothelial adherens junctions. Arterioscler Thromb Vasc Biol.

[20] Li AY, Wu YX, Gao N, Zhang JG, Meng W (2021). Expression and biological function of CMTM3 in gastric cancer. World Chin J Digestol.

[21] Yuan W, Liu B, Wang X, Li T, Han W (2017). CMTM3 decreases EGFR expression and EGF-mediated tumorigenicity by promoting Rab5 activity in gastric cancer. Cancer Lett.

[22] Burr ML, Sparbier CE, Chan YC, Williamson JC, Woods K, Beavis PA (2017). CMTM6 maintains the expression of PD-L1 and regulates anti-tumour immunity. Nature.

[23] Mezzadra R, Sun C, Jae LT, Gomez-Eerland R, de Vries E, Wu W (2017). Identification of CMTM6 and CMTM4 as PD-L1 protein regulators. Nature.

[24] Knizkova D, Pribikova M, Draberova H, Semberova T, Trivic T, Synackova A (2022). CMTM4 is a subunit of the IL-17 receptor and mediates autoimmune pathology. Nat Immunol.

[25] Choi MS, Ze EY, Park JY, Shin TS, Kim JG (2021). *Helicobacter pylori*-derived outer membrane vesicles stimulate interleukin 8 secretion through nuclear factor kappa B activation. Korean J Intern Med.

[26] Yanyan S, Yanlei G, Ting Z, Shigang D (2018). Hydrotalcite can prevent the damaging effects of *Helicobacter pylori* on gastric epithelial cells. Microsc Microanal.

[27] Cover TL, Blaser MJ (2009). *Helicobacter pylori* in health and disease. Gastroenterology.

[28] Correa P (1992). Human gastric carcinogenesis: a multistep and multifactorial process–First American Cancer Society Award Lecture on Cancer Epidemiology and Prevention. Cancer Res.

[29] Peek RJ, Blaser MJ (2002). *Helicobacter pylori* and gastrointestinal tract adenocarcinomas. Nat Rev Cancer.

[30] Dooley CP, Cohen H, Fitzgibbons PL, Bauer M, Appleman MD, Perez-Perez GI (1989). Prevalence of *Helicobacter pylori* infection and histologic gastritis in asymptomatic persons. N Engl J Med.

[31] Eftang LL, Esbensen Y, Tannaes TM, Bukholm IR, Bukholm G (2012). Interleukin-8 is the single most up-regulated gene in whole genome profiling of *H. pylori* exposed gastric epithelial cells. BMC Microbiol.

[32] Luzza F, Parrello T, Monteleone G, Sebkova L, Romano M, Zarrilli R (2000). Up-regulation of IL-17 is associated with bioactive IL-8 expression in *Helicobacter pylori*-infected human gastric mucosa. J Immunol.

[33] Crabtree JE, Wyatt JI, Trejdosiewicz LK, Peichl P, Nichols PH, Ramsay N (1994). Interleukin-8 expression in *Helicobacter pylori* infected, normal, and neoplastic gastroduodenal mucosa. J Clin Pathol.

[34] Delyria ES, Redline RW, Blanchard TG (2009). Vaccination of mice against *H. pylori* induces a strong Th-17 response and immunity that is neutrophil dependent. Gastroenterology.

[35] Purcell RV, Permain J, Keenan JI (2022). Enterotoxigenic Bacteroides fragilis activates IL-8 expression through Stat3 in colorectal cancer cells. Gut Pathog.

[36] Taguchi A, Ohmiya N, Shirai K, Mabuchi N, Itoh A, Hirooka Y (2005). Interleukin-8 promoter polymorphism increases the risk of atrophic gastritis and gastric cancer in Japan. Cancer Epidemiol Biomarkers Prev.

[37] Bockerstett KA, DiPaolo RJ (2017). Regulation of gastric carcinogenesis by inflammatory cytokines. Cell Mol Gastroenterol Hepatol.

[38] Yu XW, Xu Y, Gong YH, Qian X, Yuan Y (2011). *Helicobacter pylori* induces malignant transformation of gastric epithelial cells in vitro. APMIS.

[39] Liu JF, Guo D, Kang EM, Wang YS, Gao XZ, Cong HY (2021). Acute and chronic infection of *H. pylori* caused the difference in apoptosis of gastric epithelial cells. Microb Pathog.

[40] Boquet P, Ricci V, Galmiche A, Gauthier NC (2003). Gastric cell apoptosis and *H. pylori*: has the main function of VacA finally been identified?. Trends Microbiol.

[41] Gonciarz W, Krupa A, Hinc K, Obuchowski M, Moran AP, Gajewski A (2019). The effect of *Helicobacter pylori* infection and different *H. pylori* components on the proliferation and apoptosis of gastric epithelial cells and fibroblasts. PLoS ONE.

[42] Gonciarz W, Krupa A, Moran AP, Tomaszewska A, Chmiela M (2021). Interference of LPS *H. pylori* with IL-33-driven regeneration of caviae porcellus primary gastric epithelial cells and fibroblasts. Cells.

[43] Mnich E, Kowalewicz-Kulbat M, Sicinska P, Hinc K, Obuchowski M, Gajewski A (2016). Impact of *Helicobacter pylori* on the healing process of the gastric barrier. World J Gastroenterol.

[44] Wu J, Xu S, Zhu Y (2013). *Helicobacter pylori* CagA: a critical destroyer of the gastric epithelial barrier. Dig Dis Sci.

[45] Fujita H, Rahighi S, Akita M, Kato R, Sasaki Y, Wakatsuki S (2019). Mechanism underlying IκB kinase activation mediated by the linear ubiquitin chain assembly complex. Mol Cell Biol.

[46] Viala J, Chaput C, Boneca IG, Cardona A, Girardin SE, Moran AP (2004). Nod1 responds to peptidoglycan delivered by the *Helicobacter pylori* cag pathogenicity island. Nat Immunol.

[47] Hsin IF, Montano E, Seki E (2016). Finding a new role for NEMO: a key player in preventing hepatocyte apoptosis and liver tumorigenesis by inhibiting RIPK1. Hepatology.

[48] Kondylis V, Polykratis A, Ehlken H, Ochoa-Callejero L, Straub BK, Krishna-Subramanian S (2015). NEMO prevents steatohepatitis and hepatocellular carcinoma by inhibiting RIPK1 kinase activity-mediated hepatocyte apoptosis. Cancer Cell.

[49] Huang X, Li Y, Li X, Fan D, Xin HB, Fu M (2020). TRIM14 promotes endothelial activation via activating NF-kappaB signaling pathway. J Mol Cell Biol.

[50] Wu J, Shi Y, Pan X, Wu S, Hou R, Zhang Y (2021). SARS-CoV-2 ORF9b inhibits RIG-I-MAVS antiviral signaling by interrupting K63-linked ubiquitination of NEMO. Cell Rep.

[51] Zhou Y, Bu Z, Qian J, Cheng Y, Qiao L, Yang S (2021). Brucella melitensis UGPase inhibits the activation of NF-kappaB by modulating the ubiquitination of NEMO. BMC Vet Res.

[52] Ashida H, Kim M, Schmidt-Supprian M, Ma A, Ogawa M, Sasakawa C. A bacterial E3 ubiquitin ligase IpaH9.8 targets NEMO/IKKgamma to dampen the host NF-kappaB-mediated inflammatory response. Nat Cell Biol. 2010;12(1):66–73, 1–09. 10.1038/ncb2006 PMC3107189 20010814

[53] Medunjanin S, Putzier M, Nothen T, Weinert S, Kahne T, Luani B (2020). DNA-PK: gatekeeper for IKKgamma/NEMO nucleocytoplasmic shuttling in genotoxic stress-induced NF-kappaB activation. Cell Mol Life Sci.

[54] Huang TT, Wuerzberger-Davis SM, Wu ZH, Miyamoto S (2003). Sequential modification of NEMO/IKKgamma by SUMO-1 and ubiquitin mediates NF-kappaB activation by genotoxic stress. Cell.

[55] Wu G, Song L, Zhu J, Hu Y, Cao L, Tan Z (2018). An ATM/TRIM37/NEMO axis counteracts genotoxicity by activating nuclear-to-cytoplasmic NF-kappaB signaling. Cancer Res.

[56] Gautheron J, Courtois G (2010). "Without Ub I am nothing": NEMO as a multifunctional player in ubiquitin-mediated control of NF-kappaB activation. Cell Mol Life Sci.

[57] Kocak BT, Saribas S, Demiryas S, Yilmaz E, Uysal O, Kepil N (2020). Association between polymorphisms in HLA-A, HLA-B, HLA-DR, and DQ genes from gastric cancer and duodenal ulcer patients and cagL among cagA-positive *Helicobacter pylori* strains: the first study in a Turkish population. Infect Genet Evol.

[58] Guo Y, Zhang T, Shi Y, Zhang J, Li M, Lu F (2020). *Helicobacter pylori* inhibits GKN1 expression via the CagA/p-ERK/AUF1 pathway. Helicobacter.

[59] Lopes AI, Vale FF, Oleastro M (2014). *Helicobacter pylori* infection—recent developments in diagnosis. World J Gastroenterol.

[60] Soutto M, Chen Z, Bhat AA, Wang L, El-Rifai W (2019). Activation of STAT3 signaling is mediated by TFF1 silencing in gastric neoplasia. Nat Commun.

[61] Shi Y, Wang P, Guo Y (2019). *Helicobacter pylori*-induced DNA damage is a potential driver for human gastric cancer AGS cells. DNA Cell Biol.

